# CO_2_ Versus CH_4_ Aggregation on Trifluorobenzene: Molecular Level Characterization via Rotational Spectroscopy

**DOI:** 10.1002/anie.202513517

**Published:** 2025-09-07

**Authors:** Fan Xie, Melanie Schnell

**Affiliations:** ^1^ Hefei National Research Center for Physical Sciences at the Microscale University of Science and Technology of China Hefei Anhui 230026 China; ^2^ Deutsches Elektronen‐Synchrotron DESY Notkestr. 85 22607 Hamburg Germany; ^3^ Institut für Physikalische Chemie Christian‐Albrechts‐Universität zu Kiel Max‐Eyth‐Str. 1 24118 Kiel Germany

**Keywords:** CO_2_ and CH_4_ complexes, Ligand–gas interactions, Molecular interactions, Rotational spectroscopy

## Abstract

The cluster growth behavior of CO_2_ and CH_4_ on an aromatic ligand has been studied through the unambiguous identification of complex structures of 1,2,3‐trifluorobenzene‐(CO_2_)_1–4_ and ‐ß(CH_4_)_1–2_ using broadband rotational spectroscopy in conjunction with extensive theoretical calculations. The results reveal a contrast in the thermodynamically favorable ligand‐gas binding sites and noncovalent interactions of the two gaseous molecules on the ligand. The observation of a tunneling splitting and large centrifugal distortions indicates that CH_4_ molecules bind to the fluorinated π system via three weak hydrogen bonds without CH_4_ self‐interactions, resulting in an effective structure displaced toward the dissociation limit. Conversely, CO_2_ shows diverse and stronger intermolecular interactions with the fluorinated benzene, including F─C tetrel bonding, lone pair to π‐hole interactions, π–π stacking, and a significant contribution from CO_2_ self‐interactions. The thorough examination of ligand–gas interactions and aggregation patterns highlights the significant capacity and selectivity of the fluorinated aromatic ligand for accepting CO_2_ over CH_4_.

## Graphical abstract

What makes aromatic ligands selective for CO_2_ over CH_4_? Broadband rotational spectroscopy reveals how 1,2,3‐trifluorobenzene forms stronger F⋯C tetrel and π–π interactions with CO_2_, versus weaker CH⋯F hydrogen bonds with CH_4_, along with intriguing tunneling motions. These molecular‐level insights could benefit the design of ligands for selective gas adsorption in MOFs and related materials.

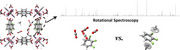

## Introduction

Carbon dioxide (CO_2_) and methane (CH_4_) are among the most potent greenhouse gases, contributing to global warming. These gases are released into the atmosphere through various processes such as fossil fuels combustion, animal husbandry, and natural gas leaks.^[^ ^1^, ^2^, ^3^ ^]^ The resulting climate change is predicted to have devastating impacts. To address this issue, measures have been taken to control the concentration of greenhouse gases in the atmosphere. One approach is the use of adsorbents for carbon capture and sequestration.^[^ ^4^ ^]^ Metal–organic frameworks (MOFs),^[^ ^5^ ^]^ zeolitic imidazolate frameworks (ZIFs),^[^ ^6^, ^7^ ^]^ and porous carbon materials^[^ ^8^, ^9^, ^10^, ^11^ ^]^ are examples of such materials that have been proposed and tested for selective gas separation and storage.

Aromatic rings such as the ligand of MOFs/ZIFs play a crucial role in the adsorption properties of these materials.^[^ ^12^ ^]^ They are the most common building blocks, providing binding sites for targeted gaseous molecules on the surface of porous materials. Extensive theoretical investigations have been performed to understand the ligand–gas interactions, such as the π–π stacking of CO_2_‐benzene complexes.^[^ ^13^, ^14^, ^15^, ^16^ ^]^ However, experimental characterizations of the structure‐specific molecular interactions, cluster growths, and nucleation dynamics at the molecular level are far from comprehensive.^[^ ^17^ ^]^ High‐resolution molecular spectroscopy is renowned for its remarkable ability to offer precise and structure‐specific characterizations of intermolecular interactions that may arise during the adsorption and cluster‐growth processes of gaseous molecules. For instance, the identification of hydrogen‐bond presence in benzene‐water^[^ ^18^ ^]^ and benzene‐ammonia^[^ ^19^ ^]^ complexes was achieved using resonance‐enhanced two‐photon ionization and microwave spectroscopy. Interestingly, this energetical priority of hydrogen‐bonded structures was found to be outpaced by the configuration that adopts an oxygen‐to‐π‐hole interaction upon full fluorination, as observed in the dimer of hexafluorobenzene‐water.^[^ ^20^ ^]^

Over the decades, researchers have expanded their investigations to include the addition of various other gaseous molecules, such as HF,^[^ ^21^ ^]^ HCl,^[^ ^22^ ^]^ HBr,^[^ ^23^ ^]^ SO_2_,^[^ ^24^ ^]^ CO,^[^ ^25^ ^]^ and N_2_,^[^ ^26^ ^]^ to benzene and substituted benzenes. The investigation of noncovalent interactions (NCI) and the corresponding binding configurations of binary complexes has yielded a wealth of experimental characterizations and valuable insights into the nature of ligand–gas interactions, thereby offering possible insights into the design and interpretation of the functionality of adsorbent materials.

In this study, we utilize 1,2,3‐trifluorobenzene (TFB), a highly polar aromatic compound, as a mimic of a functionalized ligand of porous materials to investigate its interaction strength, selectivity, and aggregation dynamics with CO_2_ and CH_4_ upon cluster growth. To achieve this, chirped‐pulse Fourier transform microwave (CP‐FTMW) spectroscopy^[^ ^27^, ^28^ ^]^ is employed in conjunction with extensive quantum‐chemical calculations. The results reveal sharp differences of CO_2_ and CH_4_ aggregation on TFB that could foster our understanding of the adsorption capacity and selectivity of porous materials contributing from ligand–gas interactions.

## Results and Discussion

### Methods

The COMPACT (compact‐passage‐acquired coherence‐technique) spectrometer^[^ ^29^ ^]^ was utilized for the measurements in the 2–8 GHz frequency range. The detailed operating principles and specifications of the instrument can be found elsewhere.^[^ ^29^, ^30^ ^]^ Here, we provide a summary of the experimental procedures. A concentration of approximately 1% CO_2_ or 5% CH_4_ was introduced into neon as the carrier gas at a backing pressure of three bars. The TFB sample (99% chemical purity) was purchased from Sigma‐Aldrich and used without further purification. The liquid TFB was placed in an internal reservoir positioned directly at the valve orifice before undergoing jet expansion at room temperature (approximately 22 °C). The gas mixture consisting of diluted TFB and CO_2_ or CH_4_ was adiabatically expanded into a vacuum chamber to generate a supersonic jet, resulting in a rotational temperature of 1 to 2 K.

To isolate the targeted rotational fingerprints of the TFB‐(CO_2_)*_n_* and TFB‐(CH_4_)*_m_* clusters from the complex global spectrum that includes TFB monomers and potential TFB hydrates, as well as CO_2_ and CH_4_ hydrates in different degrees of combination, we obtained separate spectra of pure TFB, CO_2_, and CH_4_ with slightly hydrated neon carrier gas to provide background spectra for subtraction. For each measurement, we collected around four million acquisitions in the form of a free induction decay (FID), which were subsequently averaged.

In order to facilitate the identification of binding topologies for TFB‐(CO_2_)*_n_* and TFB‐(CH_4_)*_m_* clusters, a popular approach was employed, which combines the cost‐effective conformational space exploration tool CREST^[^ ^31^, ^32^ ^]^ with quantum‐chemical calculations. Stable isomers within an energy window of 10 kJ mol^−1^ obtained using the GFN2‐xTB^[^ ^33^ ^]^ method were reoptimized using the ORCA quantum‐chemistry program package^[^ ^34^, ^35^ ^]^ at the B3LYP‐D3BJ/def2‐TZVP^[^ ^36^, ^37^, ^38^ ^]^ level of theory. Harmonic frequency calculations were performed to confirm the obtained geometries as real minima and to provide zero‐point‐energy (ZPE) corrections. The energetically low‐lying isomeric ensemble of TFB‐(CO_2_)_1–4_ and TFB‐(CH_4_)_1–4_, together with their corresponding spectroscopic constants, are presented in Tables S1 and S2.

### Spectral Assignments

**Figure 1:**
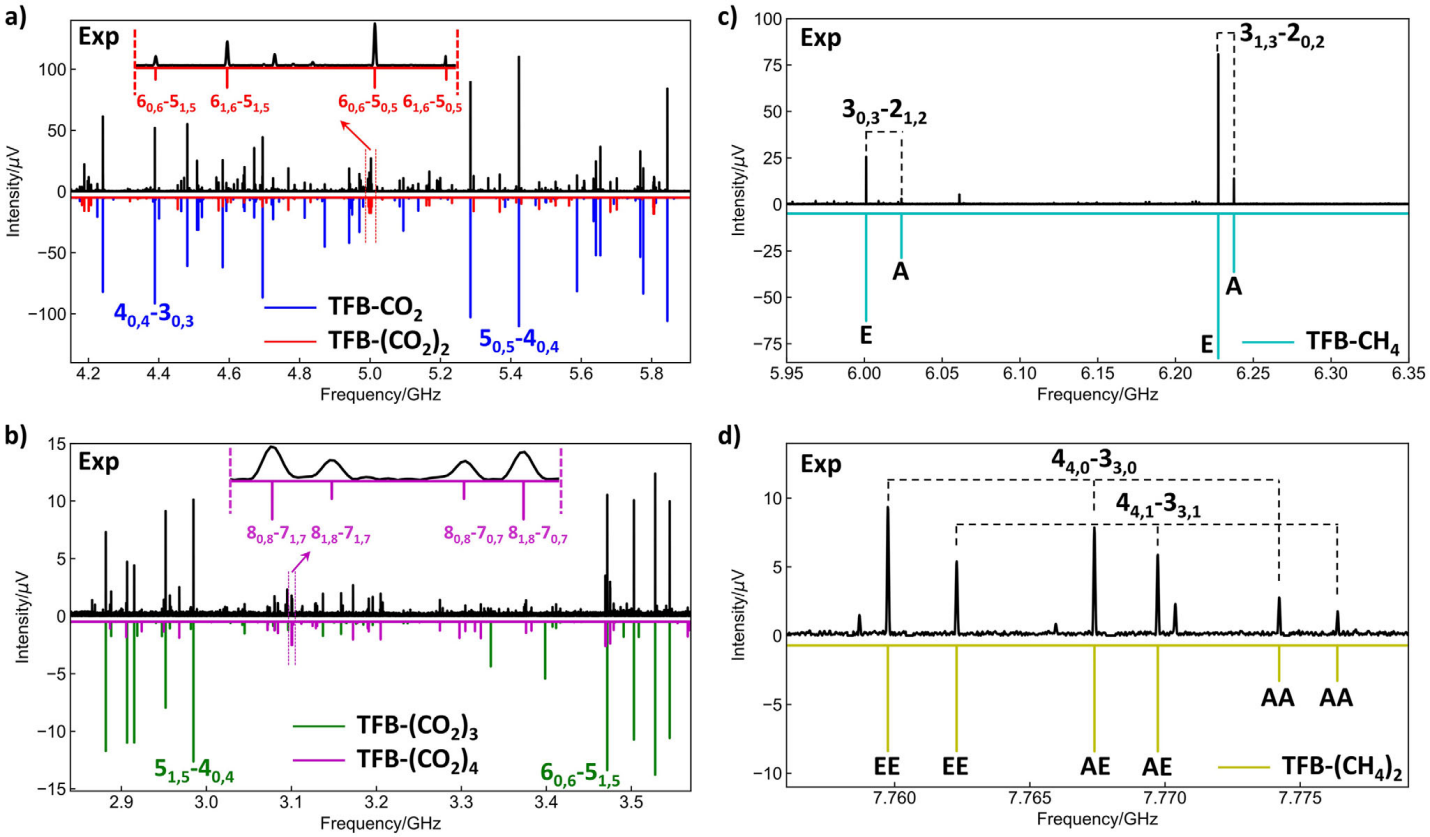
Sections of the broadband rotational spectra for TFB‐(CO_2_)_1–4_ and TFB‐(CH_4_)_1–2_ clusters. a) Broad overview of the experimental spectrum (black trace) and fitted spectra of TFB‐CO_2_ (blue trace) and TFB‐(CO_2_)_2_ (red trace). b) A zoomed‐in section of the experimental spectrum (black trace) to highlight larger TFB‐(CO_2_)_3_ and TFB‐(CO_2_)_4_ clusters together with the fitted spectra indicated with green and purple traces, respectively. The estimated relative abundances for TFB–(CO_2_)_1–4_ are approximately 67%, 20%, 8%, and 5%, respectively. c) and d) Sections of the experimental spectrum (black trace) with fitted spectra of TFB‐CH_4_ (cyan trace) and TFB‐(CH_4_)_2_ (yellow trace). The unassigned weaker transitions likely arise from higher‐order clusters or trace impurities, potentially from the TFB sample or gas delivery line. Tunneling splitting patterns arising from the torsional motion of CH_4_ are marked with A/E states, where the A state is nondegenerate, and the E state is doubly degenerate. The estimated relative abundances for TFB–(CH_4_)_1–2_ are approximately 80% and 20%, respectively. For all fitted spectra, a rotational temperature of 1 K was used in the simulation. The quantum numbers are defined using the standard nomenclature for rotational energy levels of an asymmetric top, denoted as *J*_Ka,Kc_.

**Figure 2:**
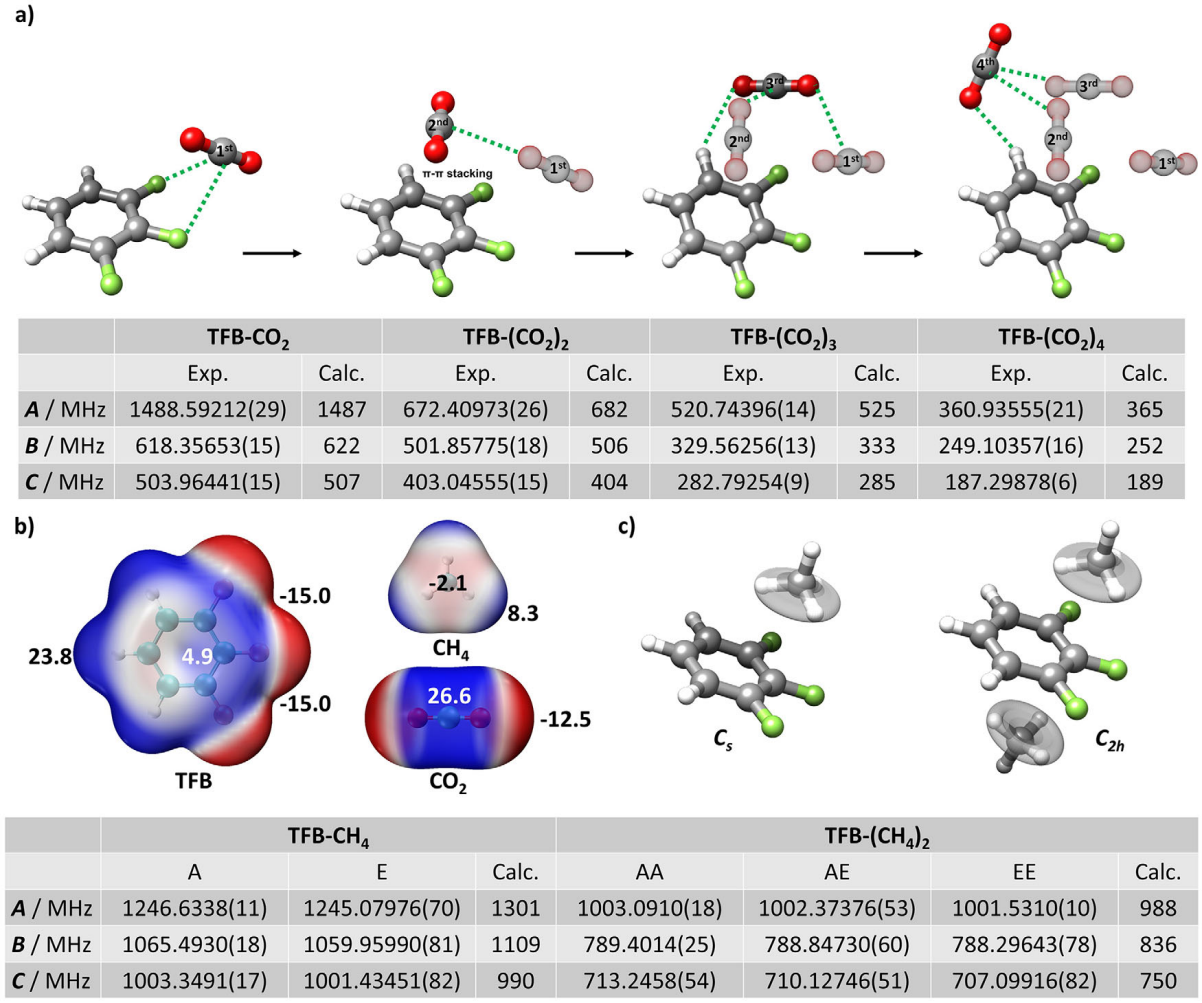
Binding configurations of the observed TFB‐(CO_2_)_1–4_ and TFB‐(CH_4_)_1–2_ complexes and comparison between their experimental and calculated rotational constants. a) The stepwise CO_2_ aggregation topologies on TFB. Dashed lines indicate the intermolecular interactions upon the addition of the n^th^ CO_2_ molecule. b) The electrostatic iso‐surface potential on 0.001e/Bohr^[^ ^3^ ^]^ iso‐surface derived from the electron densities of TFB, CH_4_, and CO_2_ monomers calculated at the B3LYP‐D3BJ/def2‐TZVP level of theory. The blue iso‐surface represents the electron‐deficient area, whereas the red iso‐surface stands for the electron‐rich area. The magnitude of the π‐hole on TFB was determined to be +4.9 kcal mol^−1^, while the σ‐hole on the carbon atom of CO_2_ was found to be +26.6 kcal mol^−1^. c) The stepwise CH_4_ aggregation topologies on TFB. For the TFB‐CH_4_ dimer, the molecular geometry adopts a *C_S_* symmetry. For the TFB‐(CH_4_)_2_, it adopts a *C_2h_* symmetry. The torsional motion of CH_4_ along one of the *C_3_* axes that leads to the observed tunneling splitting in the spectra is highlighted.

Figure 1a displays the processed experimental spectrum of TFB with CO_2_, wherein a set of strong near‐prolate a‐type transitions (blue) was observed. The corresponding structural assignment was made by comparing the fitted rotational constants and relative magnitudes of electric dipole moment components with the list of calculated candidates presented in Table S1. This comparison enabled unambiguous identification of the assigned transitions originating from the most stable TFB‐CO_2_ structure, as illustrated in Figure 2a. Further, a weaker set of transitions (red) with characteristic a‐ and b‐type quadruplets was observed. As shown in the zoomed‐in section of the spectrum, the a‐type transitions were stronger than the b‐type ones. This weaker set of transitions was assigned to TFB‐(CO_2_)_2_. The assignment of TFB‐(CO_2_)_3_ was made based on b‐type transitions (green) observed in the lower frequency range of the spectrum shown in Figure 1b. Similarly, the weakest set of transitions (purple), which contained featured b‐ and a‐type quadruplets with b‐type transitions stronger than a‐type, was assigned to TFB‐(CO_2_)_4_. For all assignments, Watson's semirigid‐rotor A‐reduction Hamiltonian^[^ ^39^ ^]^ in its *I^r^* representation was applied using PGOPHER.^[^ ^40^ ^]^ The fitted spectroscopic constants of TFB‐(CO_2_)_1–4_ are summarized in Table S3 and compared with calculations. The complete set of fitted spectroscopic constants and transition lists used for assignment are available in the uploaded PGOPHER files.^[^ ^41^ ^]^

The geometries of these identified TFB‐(CO_2_)_1–4_ complexes are shown in Figure 2a. They are the global minima of their respective isomeric pool, as presented in Table S1. This indicates the cold characteristics of our supersonic jet expansion experiments, which is consistent with previous studies where supersonic expansions were employed for the study of CO_2_‐containing molecular complexes.^[^ ^40^, ^41^, ^42^, ^43^, ^44^ ^]^ The results also show that the use of ZPE‐corrected B3LYP‐D3BJ/def2‐TZVP relative energies was sufficient to provide a reliable stability order of the TFB‐(CO_2_)_1–4_ complexes. Furthermore, the experimental and calculated rotational constants exhibit typical discrepancies of approximately 1%, as shown in Table S3, suggesting that the observed ground state structures are close to the calculated equilibrium structures.

In contrast to the experimental spectra of TFB with CO_2_, the TFB and CH_4_ mixture spectrum exhibits an intriguingly low transition density and overall intensity, despite the fact that a five times higher concentration of CH_4_ compared to that of CO_2_ was used for the measurements. As shown in Figure 1c,d, two sets of spectral signatures were assigned to the complexes of TFB‐CH_4_ and TFB‐(CH_4_)_2_, respectively. Notably, tunneling splitting transition patterns originating from the internal rotation of the CH_4_ moiety are resolved in the spectrum. These splitting patterns are similar to previous reports on CH_4_‐containing complexes, for example, symmetric tops like the CH_4_‐H_2_O^[^ ^42^ ^]^ and CH_4_‐HF^[^ ^43^ ^]^ dimers exhibit splitting patterns arising from coupled internal dynamics of CH_4_. For TFB‐CH_4_, two torsional states of the large‐amplitude motion (LAM) were assigned separately and subsequently fitted employing a semirigid‐rotor Hamiltonian. Similarly, three torsional states were assigned for TFB‐(CH_4_)_2_. The fitted spectroscopic constants and theoretical calculations are summarized in Table S4. Our results show that the A and B experimental rotational constants of TFB‐CH_4_ are about 4% smaller than the calculated values, while the B and C experimental rotational constants of TFB‐(CH_4_)_2_ are approximately 5% smaller. Furthermore, the experimental distortion constants of both TFB‐CH_4_ and TFB‐(CH_4_)_2_ are relatively large, being on the order of 10 kHz. These phenomena further suggest that CH_4_ is weakly bonded to TFB.

### Structures and Interactions

The structures of the experimentally detected TFB‐(CO_2_)_1–4_ are presented in Figure 2a. It is evident that cluster formation occurs as a stepwise CO_2_ growth on TFB. The molecular interactions involved in this aggregation process can be broadly classified into two types: TFB‐CO_2_ interactions and CO_2_ self‐binding interactions. To gain further insights into these intermolecular interactions, we have performed the electrostatic iso‐surface potential (ESP) and noncovalent interaction (NCI) analysis calculated from the B3LYP‐D3BJ/def2‐TZVP electronic configuration of isolated monomers using Multiwfn.^[^ ^44^ ^]^ As depicted in Figure 2b, the blue iso‐surface represents the electron‐deficient area, while the red iso‐surface stands for the electron‐rich area. The ESP of the TFB aromatic ring exhibits a binary pattern, with the fluorinated side of the aromatic ring being electron‐deficient and forming a partial π‐hole^[^ ^45^, ^46^, ^47^ ^]^ with the magnitude calculated to be 4.9 kcal mol^−1^, whereas the opposite side of the aromatic ring is electron‐rich. This binary feature of TFB can play a crucial role in determining the binding sites and aggregation pattern of both CO_2_ and CH_4_. For the addition of the first CO_2_ on TFB, the most favorable binding site is between the two F ends of TFB. Here, the electron‐rich F atoms bind to the electron‐deficient σ‐hole (26.6 kcal mol^−1^) of CO_2_, featuring F─C tetrel bonds.^[^ ^48^, ^49^, ^50^, ^51^, ^52^, ^53^, ^54^, ^55^, ^56^, ^57^ ^]^ In addition, one oxygen lone pair of CO_2_ binds to the electron‐deficient part of the aromatic ring, forming a lone pair to π‐hole linkage as evidenced by the NCI plots in Figure S3.

This type of linkage has been observed in other halogen‐substituted aromatic‐containing complexes.^[^ ^20^ ^]^ Furthermore, it is worth highlighting that the TFB‐CO_2_ dimer exhibits a fourfold degeneracy in its binding configuration, which arises from the inherent symmetry of the TFB monomer. This symmetry results in four energetically equivalent binding sites for the first CO_2_ molecule.

Interestingly, the subsequent addition of a second CO_2_ molecule does not preferentially bind to any of the three remaining binding sites. Instead, this second CO_2_ binds to the top of the TFB‐π orbital, where π–π stacking dominates the TFB‐CO_2_ interactions. In addition, the CO_2_ self‐binding interactions also contribute significantly to the overall stability of the complex, resulting in the formation of a T‐shaped CO_2_ dimer.^[^ ^58^ ^]^ As the addition of the third and fourth CO_2_ molecules progresses, the CO_2_ self‐binding interactions become increasingly dominant in determining the complexation topologies. Notably, in all observed TFB‐CO_2_ complexes, only one quarter of the TFB surface was utilized for CO_2_ binding. These configurations promote compact CO_2_ aggregation on TFB, thereby maximizing intermolecular interactions, especially CO_2_ self‐interactions.

The identified structure of the TFB‐CH_4_ dimer is presented in Figure 2c, which features three weak hydrogen bonds as the major contribution to the noncovalent interactions. This structure involves two electron‐deficient H atoms of CH_4_ and three electron‐rich F atoms of TFB, resulting in two bifurcated hydrogen bonds. Moreover, another hydrogen bond is formed between the electron‐rich portion of the aromatic ring and one hydrogen atom of CH_4_. This binding configuration gives rise to a *C_s_* symmetry of the TFB‐CH_4_ dimer. Notably, there are two equivalent binding sites on TFB for interacting with CH_4_, namely the top and bottom sides of the aromatic ring. Interestingly, the second CH_4_ molecule attaches to the other equivalent side on TFB via another three weak hydrogen bonds identical to the addition of the first CH_4_, resulting in the higher *C_2h_* symmetry of TFB‐(CH_4_)_2_. This is in sharp contrast to the cluster growth of CO_2_, as self‐aggregation of CH_4_ was not observed at this level of complexation.

To gain further insights into the evolution of interaction strength and the factors contributing to it upon the stepwise CO_2_ and CH_4_ aggregation on TFB, an energy analysis was performed using symmetry‐adapted perturbation theory (SAPT)^[^ ^59^ ^]^ intermolecular binding energy decomposition calculations via the PSI4 software package.^[^ ^60^ ^]^ The focus was on binary binding energies Δ*E*_total_, which represents the interaction strength of the n^th^ CO_2_ or CH_4_ molecule on TFB. For instance, Δ*E*_total_ of [TFB‐(CO_2_)]‐CO_2_ was calculated by dividing the observed TFB‐(CO_2_)_2_ structure into two partners: [TFB‐(CO_2_)] and the second CO_2_. These binding energies were then broken down into four physically meaningful terms, namely electrostatics, induction, dispersion, and exchange, using the SAPT2 + 3/aug‐cc‐pVDZ level of theory, including δMP2 corrections.^[^ ^61^ ^]^ The results are summarized in Table 1. These support that CO_2_ has a stronger interaction with TFB than CH_4_. Dispersion interactions were found to dominate the attractive contributions in both CO_2_ and CH_4_ cases, while the electrostatic contribution in CO_2_ aggregation on TFB was higher than that of CH_4_, owing to the greater polarity of C═O than C─H. Moreover, the analysis reveals an increasing trend in the evolution of the interaction strength over the course of CO_2_ cluster growth on TFB, owing to the CO_2_ self‐aggregation interactions. Such effects were not observed in the case of CH_4_ aggregation on TFB. The binding energy contributions from the addition of the first and second CH_4_ on TFB are nearly identical.

**Table 1:** Binary binding energy decomposition calculated using SAPT2 + 3/aug‐cc‐pVDZ with δMP2 corrections in kJ mol^−1^. Each of the observed TFB‐(CO_2_)_1–4_ and ‐(CH_4_)_1–2_ complexes was divided into two structural components as indicated in the left row. The total binding energy Δ*E*_total_ represents the n^th^ CO_2_ or CH_4_ interaction energies with TFB.

Binary structures	Δ*E*_Electrostatic_	Δ*E*_Induction_	Δ*E*_Dispersion_	Δ*E*_Exchange_	Δ*E*_total_
TFB‐CO_2_	−8.2 (37.6%)	−1.1 (5.1%)	−12.5 (57.3%)	12.3	−9.5
[TFB‐(CO_2_)]‐CO_2_	−10.7 (31.5%)	−1.9 (5.5%)	−21.3 (62.9%)	20.2	−13.5
[TFB‐(CO_2_)_2_]‐CO_2_	−15.1 (40.0%)	−1.8 (4.8%)	−20.8 (55.2%)	21.8	−15.9
[TFB‐(CO_2_)_3_]‐CO_2_	−12.2 (38.3%)	−1.7 (5.2%)	−18.0 (56.4%)	17.7	−14.2
TFB‐CH_4_	−4.5 (25.1%)	−0.5 (2.8%)	−12.8 (72.1%)	10.9	−6.9
[TFB‐(CH_4_)]‐CH_4_	−4.4 (24.9%)	−0.5 (2.8%)	−12.8 (72.4%)	10.8	−6.9

To assess whether these selective trends hold for other MOF/ZIF‐relevant aromatic ligands, we performed analogous SAPT calculations on binary complexes of representative aromatics; these include benzene, 1‐fluorobenzene, hexafluorobenzene, trifluorotoluene, aniline, and nitrobenzene with a single CO_2_ or CH_4_ molecule (Table S9 and Figure S4). All ligands preferentially bind CO_2_ over CH_4_. Among them, aniline shows the strongest CO_2_ interaction, driven by a strong tetrel bond between the CO_2_ carbon and the amine group. Increasing fluorination does not necessarily enhance CO_2_ binding; although it introduces additional diverse noncovalent interactions (e.g., F─C tetrel bonding), it can also weaken π–π stacking, thus reducing dispersion interactions. Trifluorotoluene achieves an optimal balance by maintaining π–π interactions while enabling tetrel bonding, resulting in the highest CO_2_‐over‐CH_4_ selectivity among the fluorinated aromatics studied.

### CH_4_ Dynamics on TFB

The assignments of multiple torsional states in the spectra of the TFB‐(CH_4_)_1–2_ complexes suggest that CH_4_ undergoes internal rotation motions with respect to the TFB monomer. As illustrated in Figure S1, there are five distinct internal rotation pathways: two motions that rotate around one of the *C_2_* symmetry axes of CH_4_ and three motions that rotate around one of the *C_3_* symmetry axes. The corresponding potential energy scans for these motions were calculated at the B3LYP‐D3BJ/def2‐TZVP level of theory. The coexistence of these rotations would lead to complex spectral splitting patterns. However, at the resolution limit (25 kHz) of the CP‐FTMW spectrometer, we observe relatively simple splitting patterns—two states for TFB‐CH_4_ and three states for TFB‐(CH_4_)_2_. These splitting patterns resemble those typically seen for internal rotation of methyl tops. In the case of TFB‐(CH_4_)_2_, the pattern is akin to two non‐coupling equivalent methyl tops. Therefore, we conclude that the observed splitting pattern likely arises from the *C_3_* torsional motion associated with the lowest calculated energy barrier (0.7 kJ mol^−1^), where the torsional axis lies within the mirror plane of the TFB‐(CH_4_)_1–2_ complexes, as shown in Figure 2c. Despite extensive attempts, a meaningful global fit for TFB‐(CH_4_)_1–2_ using internal rotation programs such as XIAM^[^ ^62^ ^]^ was not achieved. This difficulty likely arises from the fact that the internal rotation axis of CH_4_ with respect to TFB is not constrained by a covalent bond but might be flexible due to the weakly‐bound character of the complex.

**Figure 3:**
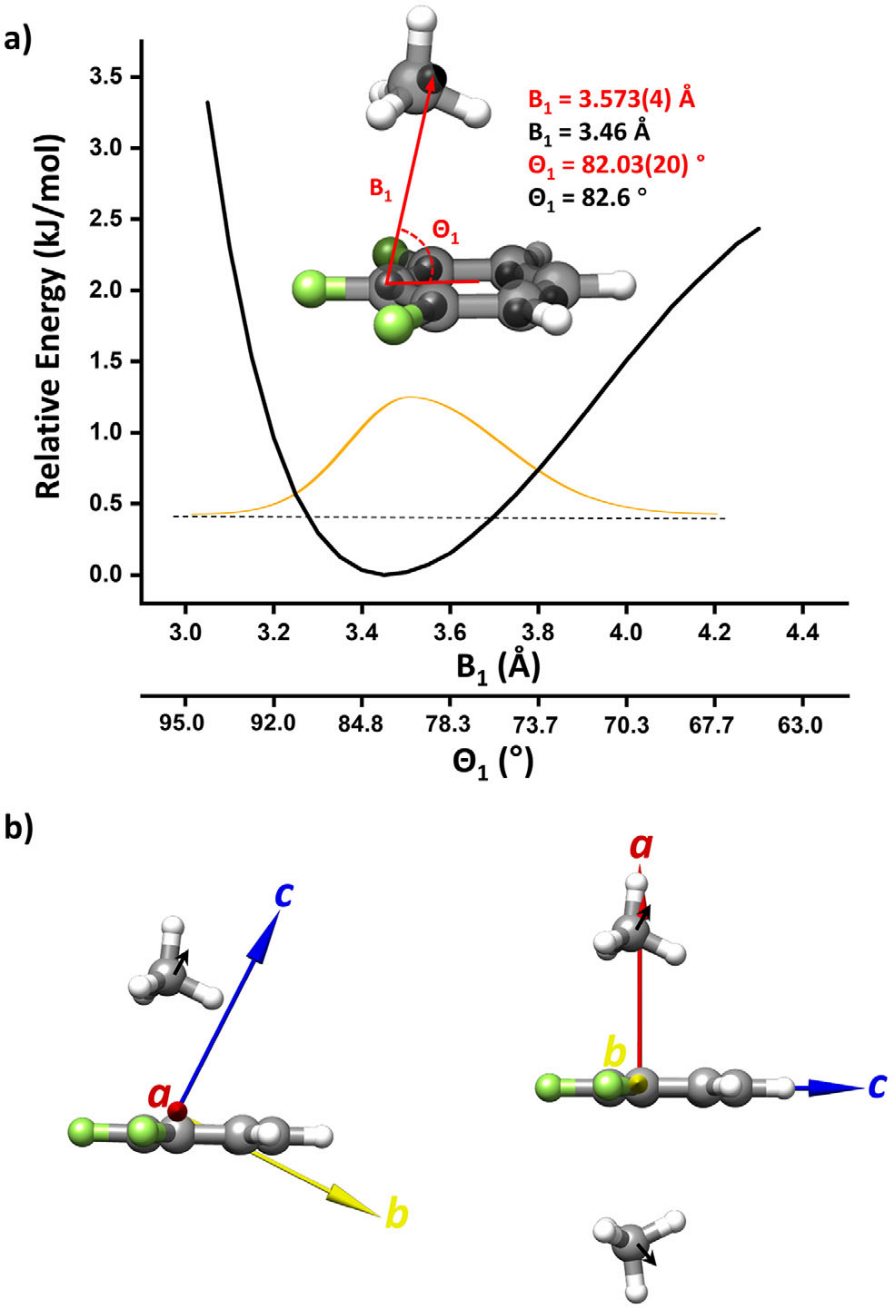
a) The relaxed energy scan profile as a function of the distance between CH_4_ and TFB calculated at the B3LYP‐D3BJ/def2‐TZVP level of theory. Two coordinates, B_1_ and Θ_1_, ranging from 3.0 to 4.4 Å and 95° to 63°, respectively, were used to characterize the large amplitude motion. In the molecular structure of the TFB‐CH_4_ dimer, the potential well's minimum structure is shown, with B_1_ and Θ_1_ adopting values of 3.46 Å and 82.6°, respectively. The Kraitchman coordinates derived from the analysis of the ^13^C singly substituted isotopologues are represented by solid black balls. The corresponding coordinates obtained through rotational spectroscopy, B_1_ and Θ_1_, are determined to be 3.573(4) Å and 82.03(20)°, respectively. b) The minimum structures of the observed TFB‐CH_4_ and TFB‐(CH_4_)_2_ complexes plotted within the principal axis system. The black arrow on the CH_4_ molecules indicates the direction of effective displacement from the minimum structure caused by the large‐amplitude motions.

Furthermore, the substantial differences between the observed torsional states structures and the calculated equilibrium structures for TFB‐(CH_4_)_1–2_, as shown in Table S4, suggest the presence of an additional large amplitude motion beyond the internal rotation of CH_4_. This leads to less compact, effective structures compared to the calculated minima structures. To gain further information on the internal dynamics of CH_4_ on TFB from experiments, we searched for the spectra of the singly ^13^C‐substituted isotopologues of the TFB‐CH_4_ dimer in natural abundances. These results are listed in Tables S5 and S6 and in Figure S2. The corresponding Kraitchman coordinates of the carbon atoms, along with the optimized minimum structure from quantum chemistry, are presented in Figure 3a. The effective structure of the dimer retains its *C_S_* symmetry, with the C atom of CH_4_ situated on the mirror plane. However, a noticeable difference arises in the position of CH_4_ on the symmetry plane, as compared to the calculated minimum structure. Specifically, the effective position of CH_4_ is further away from the F substitution end of the aromatic ring. This structural variation can be described by two coordinates, B_1_ and Θ_1_, as defined in Figure 3a. To gain insight into the energy profile of these two degrees of freedom, we performed a relaxed energy survey following the motion at the B3LYP‐D3BJ/def2‐TZVP level of theory. A characteristic anharmonic potential well was revealed, as shown in Figure 3a. The nuclei wavefunction of the TFB‐CH_4_ dimer, representing the spatial probability distribution of CH_4_, is asymmetric with larger integral on the right side. This provides a plausible and consistent explanation for the observed difference between the calculated minimum structure and the experimental effective structure.

As illustrated in Figure 3b, the large amplitude motion of CH_4_ results in its effective displacement from the principal axes *a* and *b*, which leads to smaller experimental A and B rotational constants compared to the quantum‐chemical equilibrium values. Similarly, in TFB‐(CH_4_)_2_, the motion of both CH_4_ molecules away from the *b* and *c* principal axes leads to a reduction in the experimental rotational constants of B and C. For comparison, we also present the Kraitchman structure of the TFB‐CO_2_ dimer in Figure S2. The effective structure is well matched with the calculated minimum, which is consistent with the 1% discrepancies (Table S3) of a typical rigid complex.

## Conclusion

The rotational spectroscopic identification of the (CO_2_)_1–4_ and (CH_4_)_1–2_ aggregation pattern on TFB was performed with the aid of extensive theoretical calculations. The assigned six parent species are the global minima of their respective isomeric pool, revealing the thermodynamically preferred ligand‐to‐gas‐binding sites. The interaction of CO_2_ with the fluorinated aromatic ring is shown to be stronger than that of CH_4_ by the evaluation of experimental rotational constants, centrifugal distortion constants, and binding energy decomposition analysis. Furthermore, one quarter of the TFB surface is capable of accepting the growing CO_2_ cluster up to the CO_2_ tetramer with the configuration stabilized by CO_2_ self‐interaction, whereas one TFB molecule is observed to be capable of accepting two CH_4_ molecules separately on the two faces of the aromatic ring. This indicates much higher adsorption capacities of aromatic ligands for CO_2_ compared to CH_4_.

While TFB serves as a chemically relevant mimic of fluorinated aromatic ligands in MOFs/ZIFs, certain limitations must be acknowledged when extrapolating to extended frameworks. In particular, the absence of metal–carboxylate coordination in TFB precludes electronic effects induced by metal node conjugation, which modulate π‐electron distribution and alter gas‐binding strength and orientation in actual MOFs. Additionally, the isolated nature of TFB does not reflect the steric constraints present in porous materials, where neighboring and confined environments can restrict binding site accessibility or influence aggregation geometry. Nevertheless, the noncovalent interaction motifs identified here—such as F─C tetrel bonding, lone‐pair to π‐hole interactions, and CH─F hydrogen bonding—are localized and intrinsic in nature. These interactions are likely to persist in extended systems and can serve as transferable building blocks for interpreting gas selectivity.

Among the fluorinated aromatics studied, trifluorotoluene is predicted to exhibit the strongest intrinsic CO_2_‐over‐CH_4_ binding selectivity, attributed to its dual capacity for tetrel bonding and π–π interactions. This underscores CF_3_ substitution as a promising motif for selective CO_2_ recognition in the low‐loading, single‐molecule regime. However, translating these findings to high‐coverage conditions, multi‐guest co‐adsorption, or frameworks with more elaborate ligand topologies will require further systematic investigation. In particular, the interplay between fluorination patterns, steric accessibility, and cooperative interactions in complex ligands remains an open challenge for achieving selective and efficient gas capture.

## Conflict of Interests

The authors declare no conflict of interest.

## Supporting information



Supporting Information

## Data Availability

The data that support the findings of this study are openly available in Zenodo at https://doi.org/[10.5281/zenodo.14830615], reference number 14830616.
