# Supplementary material for: CO2 Versus CH4 Aggregation on Trifluorobenzene: Molecular Level Characterization via Rotational Spectroscopy
Source: Angew Chem Int Ed Engl. 2025 Sep 7;64(43):e202513517. doi: 10.1002/anie.202513517 (PMC12535381; doi:10.1002/anie.202513517)
Supplement: Supplementary file 1 — Supporting Information [file ANIE-64-e202513517-s001.pdf]

Supporting Information
©Wiley-VCH 2023
69451 Weinheim, Germany

CO_2 *versus* CH_4 Aggregation on Trifluorobenzene: Molecular Level Characterization via Rotational Spectroscopy

Fan Xie*, Melanie Schnell*

[a] Prof. Dr. Fan Xie
Hefei National Research Center for Physical Sciences at the Microscale, University of Science and Technology of China, Hefei, Anhui, 230026, China
Deutsches Elektronen-Synchrotron DESY, Notkestr. 85, 22607 Hamburg, Germany
E-mail: xiefan@ustc.edu.cn

[b] Prof. Dr. Melanie Schnell
Deutsches Elektronen-Synchrotron DESY, Notkestr. 85, 22607 Hamburg, Germany
Institut für Physikalische Chemie, Christian-Albrechts-Universität zu Kiel, Max-Eyth-Str. 1, 24118 Kiel, Germany
E-mail: melanie.schnell@desy.de

Table of Contents

Table S1. Relative ZPE corrected (ΔD_0) energies and raw dissociation energies (ΔD_e) in kJ mol^{-1}, rotational constants in MHz, and electric dipole moment components in Debye of the TFB-$(CO_2)_n$, n=1-4, isomers, optimized at the B3LYP-D3(BJ)/def2-TZVP level of theory. Observed structures are indicated with red……………………………3

Table S2. Relative ZPE corrected (ΔD_0) energies and raw dissociation energies (ΔD_e) in kJ mol^{-1}, rotational constants in MHz, and electric dipole moment components in Debye of the TFB-$(CH_4)_n$, n=1-4, isomers, optimized at the B3LYP-D3(BJ)/def2-TZVP level of theory. Observed structures are indicated with red.………………………..6

Table S3. Comparison between experimentally determined and calculated rotational parameters of TFB-$(CO_2)_n$, (n = 1-4). *A*, *B*, and *C* are the rotational constants, μ_a, μ_b, and μ_c are the electric dipole-moment components. σ is the root-mean-square deviation of the fit, N is the number of rotational transitions included in the fit. The calculated spectroscopic constants of the minimum structures are optimized at the B3LYP-D3(BJ)/def2-TZVP level of theory………………………………………………………………………………………………………......................7

Table S4. Comparison between experimentally determined and calculated rotational parameters of TFB-$(CH_4)_n$, (n = 1-2). *A*, *B*, and *C* are the rotational constants, μ_a, μ_b, and μ_c are the electric dipole-moment components. σ is the root-mean-square deviation of the fit, N is the number of rotational transitions included in the fit. The calculated spectroscopic constants of the minimum structures are optimized at the B3LYP-D3(BJ)/def2-TZVP level of theory………………………………………………………………………………………………………......................7

Figure S1. The possible internal rotation motions of CH_4 on TFB………………………………………….………8

Table S5. The fitted rotational constants of TFB-CH_4 dimer ^{13}C isotopologues. The distortion constants are adopted from the parent fitting……………………………………………………………………………………………………9

Table S6. The Kraitchman coordinates of TFB-CH_4 dimer ^{13}C isotopologues with the sign adopted from the optimized minimum structure……………………………………………………………………………………...9

Table S7. The fitted rotational constants of TFB-CO_2 dimer ^{13}C isotopologues. The distortion constants are adopted from the parent fitting……………………………………………………………………………………………………9

Table S8. The Kraitchman coordinates of TFB-CO_2 dimer ^{13}C with the sign adopted from the optimized minimum structure……..9

Figure S2. The Kraitchman structure and atom number label of TFB-CH_4 and TFB-CO_2 dimers………………......10

Figure S3. NCI plots of the observed TFB-$(CO_2)_n$ and TFB-$(CH_4)_m$ complexes.……………………………….......10

Figure S4. The optimized structures of representative aromatics-gas complexes, calculated at B3LYP-D3(BJ)/def2-TZVP level of theory.…………………………………………………………………………………….……....11

Table S9. Binary binding energy decomposition of representative aromatics-gas complexes, calculated using SAPT2+3/aug-cc-pVDZ with δMP2 corrections in kJ/mol. The geometries correspond to the global minima of aromatics-gas binary complexes, each selected from their respective isomeric pools……………………………….11

Table S1. Relative ZPE corrected (ΔD_0) energies and raw dissociation energies (ΔD_e) in kJ mol^{-1}, rotational constants in MHz, and electric dipole moment components in Debye of the TFB-$(CO_2)_n$, n=1-4, isomers, optimized at the B3LYP-D3(BJ)/def2-TZVP level of theory. Observed structures are indicated with red.

TFB-CO_2								
	ΔD_0[a]	ΔD_e	A	B	C	$\|\mu_a\|$	$\|\mu_b\|$	$\|\mu_c\|$
**1**	**0.0**	**0.0**	**1487**	**622**	**507**	**2.3**	**1.8**	**0.9**
2	1.8	2.1	1967	438	358	0.1	2.8	0.0
3	4.9	5.6	1861	313	268	2.9	1.4	0.0
**TFB-$(CO_2)_2$**								
	ΔD_0[a]	ΔD_e	A	B	C	$\|\mu_a\|$	$\|\mu_b\|$	$\|\mu_c\|$
**1**	**0.0**	**0.0**	**682**	**506**	**404**	**2.1**	**1.7**	**0.9**
2	0.9	0.8	678	507	390	0.0	2.3	1.4
3	1.3	1.4	812	426	313	1.1	2.8	0.7
4	3.7	4.5	694	441	375	2.6	0.0	1.8
5	3.7	4.2	607	475	323	0.0	3.2	0.0
6	3.7	4.0	932	296	283	2.3	0.4	1.5
7	3.9	4.3	938	340	298	0.4	2.3	1.6
8	5.6	6.3	916	280	232	1.1	2.6	0.9
9	8.5	9.6	1214	191	182	3.1	1.5	0.0
**TFB-$(CO_2)_3$**								
	ΔD_0[a]	ΔD_e	A	B	C	$\|\mu_a\|$	$\|\mu_b\|$	$\|\mu_c\|$
**1**	**0.0**	**0.0**	**525**	**333**	**285**	**0.8**	**2.8**	**0.1**
2	1.1	1.3	414	391	348	1.8	1.7	1.4
3	1.8	1.9	463	357	316	0.5	2.7	0.0
4	2.9	3.5	455	344	323	2.6	0.7	1.6
5	3.5	4.2	498	326	286	1.0	2.5	1.1
6	3.5	3.9	463	366	263	1.7	2.0	0.8
7	3.7	4.3	530	300	263	0.7	2.3	1.7
8	4.0	4.6	501	330	225	0.9	2.7	1.4
9	4.1	4.4	601	268	246	1.5	2.0	0.7
10	4.6	5.3	457	355	240	0.4	2.9	1.2
11	4.9	5.5	413	388	264	3.0	1.3	1.1
12	5.2	5.8	490	330	270	2.8	1.4	0.7
13	5.3	6.3	485	349	279	3.1	0.4	1.0
14	5.8	6.5	540	276	244	1.4	2.7	0.3
15	5.8	6.5	503	284	230	0.2	1.1	2.3
16	5.8	6.5	477	309	224	0.1	2.4	1.7
17	6.0	6.8	452	331	279	2.4	1.6	1.6
18	6.1	6.8	628	254	213	2.7	0.0	1.9
19	6.1	7.0	434	350	285	3.0	0.9	1.1
20	6.5	7.6	442	332	272	1.2	2.6	1.1
21	6.6	7.6	461	356	238	3.5	0.0	0.1
22	7.1	8.1	696	238	200	3.1	0.2	1.2
23	7.2	8.3	431	329	272	1.5	2.5	1.0
24	7.3	8.3	556	265	206	0.5	3.2	0.2

25	7.4	8.4	512	299	223	0.1	2.2	2.0
26	7.6	8.5	716	217	183	1.9	2.5	0.9
27	8.9	10.0	597	251	238	3.0	0.6	1.1
28	9.1	10.4	680	191	164	0.6	2.8	0.7
				**TFB-$(CO_2)_4$**				
	ΔD_0[a]	ΔD_e	A	B	C	|μ_a|	|μ_b|	|μ_c|
**1**	**0.0**	**0.0**	**365**	**252**	**189**	**1.5**	**2.3**	**0.1**
2	0.8	1.1	333	276	220	0.1	2.6	1.6
3	1.5	1.7	363	245	224	2.0	1.8	0.2
4	1.8	2.2	342	268	249	2.2	0.4	1.8
5	1.8	2.2	381	244	208	1.7	1.6	1.9
6	1.9	2.1	440	208	197	0.6	2.7	0.6
7	2.1	2.3	372	244	230	0.1	0.2	2.9
8	2.4	2.9	330	282	242	1.2	2.8	0.7
9	2.4	2.9	347	262	239	0.5	0.9	2.7
10	2.5	2.8	379	228	204	0.3	2.4	1.4
11	2.7	3.2	370	247	236	1.6	1.1	2.2
12	2.9	3.7	344	271	219	0.7	2.6	1.5
13	3.0	3.7	343	273	220	0.7	2.6	1.5
14	3.0	3.6	389	242	200	1.2	2.6	0.8
15	3.0	3.6	315	282	205	2.2	1.6	0.7
16	3.0	3.6	314	283	206	2.3	1.6	0.7
17	3.1	3.6	314	284	207	2.3	1.5	0.7
18	3.2	3.9	336	276	225	1.8	1.8	1.6
19	3.4	3.8	313	305	206	3.2	1.2	0.4
20	3.9	4.4	317	280	239	2.4	1.3	1.1
21	3.9	4.5	322	279	247	3.6	0.0	0.0
22	4.1	4.9	304	282	232	1.3	3.0	0.8
23	4.2	4.9	420	213	208	2.3	0.8	2.2
24	4.5	5.3	343	258	227	2.8	0.2	0.6
25	4.7	5.4	450	200	184	2.2	1.9	1.3
26	4.9	5.8	358	261	229	3.5	0.2	0.1
27	4.9	5.8	359	260	229	3.5	0.2	0.1
28	4.9	5.6	362	255	218	3.2	0.6	1.0
29	5.0	5.7	386	220	207	1.0	0.4	2.8
30	5.1	5.8	329	254	235	2.9	0.1	1.1
31	5.1	5.8	410	216	194	3.1	0.7	1.2
32	5.1	5.8	419	213	193	3.0	0.8	1.3
33	5.2	6.1	431	201	197	1.5	2.2	1.5
34	5.4	6.2	332	251	224	2.1	1.0	2.0
35	5.4	6.5	332	245	237	3.3	0.0	0.2
36	5.6	6.5	314	274	231	2.3	1.2	1.8
37	5.6	6.5	313	274	231	2.3	1.2	1.8
38	5.6	6.5	332	240	212	0.5	1.1	2.7

39	5.6	6.5	413	206	166	0.2	2.9	1.2
40	5.8	6.7	327	254	223	2.0	1.8	1.7
41	5.9	6.8	371	251	198	2.2	1.9	1.5
42	5.9	6.8	358	259	206	2.1	2.0	1.2
43	5.9	6.7	335	253	196	1.0	2.5	1.7
44	5.9	6.9	352	265	218	2.3	1.8	1.5
45	6.0	7.0	306	280	223	2.5	0.3	1.7
46	6.0	7.1	311	276	223	0.8	2.9	1.1
47	6.2	7.1	343	272	192	1.5	2.6	1.1
48	6.2	7.2	410	215	206	2.3	0.0	2.2
49	6.3	7.3	327	257	198	0.5	2.6	1.5
50	6.6	7.7	306	263	236	1.5	2.5	1.6
51	6.6	7.7	306	264	235	1.5	2.6	1.5
52	7.2	8.6	343	242	219	0.0	2.9	0.0
53	7.2	8.6	342	243	219	0.0	2.9	0.0
54	7.2	8.4	400	210	181	0.7	2.9	0.9
55	7.3	8.3	366	227	188	1.3	2.9	1.1
56	7.4	8.5	330	261	200	0.2	3.1	0.7
57	7.5	8.9	326	269	229	3.4	0.6	0.4
58	7.6	8.9	321	267	226	3.4	0.7	0.1
59	8.1	9.2	319	252	224	2.6	1.2	1.7

[a] Relative ZPE corrected energies are referenced to the global minimum structure at B3LYP-D3(BJ)/def2-TZVP

Table S2. Relative ZPE corrected (ΔD_0) energies and raw dissociation energies (ΔD_e) in kJ mol^{-1}, rotational constants in MHz, and electric dipole moment components in Debye of the TFB-$(CH_4)_n$, n=1-4, isomers, optimized at the B3LYP-D3(BJ)/def2-TZVP level of theory. Observed structures are indicated with red.

**TFB-CH_4**								
	ΔD_0[a]	ΔD_e	A	B	C	$\|\mu_a\|$	$\|\mu_b\|$	$\|\mu_c\|$
**1**	**0.0**	**0.0**	**1301**	**1109**	**990**	**0.0**	**2.8**	**0.6**
2	2.7	3.4	1858	740	531	2.5	1.6	0.1
3	2.8	3.8	2215	723	547	0.9	2.7	0.0
4	2.8	3.4	1820	739	527	2.7	1.3	0.0
5	3.0	3.8	2215	725	548	0.9	2.7	0.0
6	3.3	4.8	1735	685	493	3.0	0.0	0.1
**TFB-$(CH_4)_2$**								
	ΔD_0[a]	ΔD_e	A	B	C	$\|\mu_a\|$	$\|\mu_b\|$	$\|\mu_c\|$
**1**	**0.0**	**0.0**	**988**	**836**	**750**	**0.0**	**0.0**	**2.8**
2	2.8	3.2	1123	593	527	2.7	1.3	0.0
3	2.9	3.3	1129	584	519	2.6	1.2	0.5
**TFB-$(CH_4)_3$**								
	ΔD_0[a]	ΔD_e	A	B	C	$\|\mu_a\|$	$\|\mu_b\|$	$\|\mu_c\|$
1	0.0	0.0	804	639	550	0.4	1.4	2.4
2	0.0	-0.2	883	577	509	0.5	0.3	2.7
3	0.1	-0.1	817	635	542	0.6	1.0	2.5
4	0.5	0.8	868	541	511	1.5	1.0	2.1
5	0.6	0.8	865	536	512	1.6	1.1	2.0
6	1.3	1.6	760	517	467	2.6	0.0	1.3
7	1.3	1.7	763	518	468	2.6	0.3	1.3
8	1.4	1.1	833	557	471	0.0	1.5	2.2
9	1.8	2.0	810	570	449	2.4	0.3	1.4
10	1.9	1.6	883	505	433	2.1	2.0	0.1
11	2.1	1.7	976	477	448	0.9	0.9	2.4
12	3.3	3.7	911	444	380	2.0	2.1	0.3
13	4.8	5.4	1037	401	360	0.0	2.7	0.1
**TFB-$(CH_4)_4$**								
	ΔD_0[a]	ΔD_e	A	B	C	$\|\mu_a\|$	$\|\mu_b\|$	$\|\mu_c\|$
1	0.0	0.0	684	463	445	0.3	0.1	2.7
2	0.0	0.3	650	485	445	0.6	0.0	2.7
3	0.2	0.6	671	474	409	0.6	0.7	2.6
4	0.4	0.5	673	457	417	1.3	0.8	2.3
5	0.4	0.1	584	555	385	0.8	0.0	2.6
6	0.6	0.5	727	463	396	0.8	0.8	2.5
7	0.6	0.3	801	406	380	0.0	0.0	2.7
8	0.7	0.3	576	538	382	0.0	0.8	2.6
9	0.8	0.3	810	408	379	0.1	0.1	2.7
10	0.8	0.6	631	484	399	1.9	2.0	0.4
11	1.0	0.8	679	410	390	1.8	1.9	1.0

12	1.1	1.3	719	407	392	0.6	2.2	1.5
13	1.2	1.3	697	416	390	0.7	2.3	1.3
14	1.2	1.2	587	527	394	1.1	1.1	2.3
15	1.2	0.8	686	458	368	0.8	0.3	2.5
16	1.3	0.8	577	540	379	0.0	2.0	1.8
17	1.3	1.1	627	486	406	1.2	0.8	2.4
18	1.8	1.8	685	413	406	0.9	1.0	2.3
19	2.1	1.7	738	377	353	1.8	2.2	0.1
20	2.8	2.4	750	404	381	0.7	2.6	0.7
21	3.1	2.9	682	389	360	1.3	1.1	2.2
22	3.8	3.8	808	351	339	0.1	2.4	1.2

[a] Relative ZPE corrected energies are referenced to the global minimum structure at B3LYP-D3(BJ)/def2-TZVP

Table S3. Comparison between experimentally determined and calculated rotational parameters of TFB-$(CO_2)_n$, (n = 1-4). *A*, *B*, and *C* are the rotational constants, μ_a, μ_b, and μ_c are the electric dipole-moment components. σ is the root-mean-square deviation of the fit, N is the number of rotational transitions included in the fit. The calculated spectroscopic constants of the minimum structures are optimized at the B3LYP-D3(BJ)/def2-TZVP level of theory.

	TFB-CO_2	Calc.	Diff. / %	TFB-$(CO_2)_2$	Calc.	Diff. / %	TFB-$(CO_2)_3$	Calc.	Diff. / %	TFB-$(CO_2)_4$	Calc.	Diff. / %
A / MHz	1488.59212(29)	1487	+0.1	672.40973(26)	682	-1.4	520.74396(14)	525	-0.8	360.93555(21)	365	-1.1
B / MHz	618.35653(15)	622	-0.6	501.85775(18)	506	-0.8	329.56256(13)	333	-1.0	249.10357(16)	252	-0.8
C / MHz	503.96441(15)	507	-0.6	403.04555(15)	404	-0.2	282.79254(9)	285	-0.8	187.29878(6)	189	-0.9
|Dipole comp.|	$\mu_a > \mu_b > \mu_c$	2.3/ 1.9/ 0.9	-	$\mu_a > \mu_b > \mu_c$	2.1/ 1.7/ 0.9	-	$\mu_b > \mu_a$, no μ_c	0.8/ 2.8/ 0.	-	$\mu_b > \mu_a$, no μ_c	1.5/ 2.3/ 0.1	-
N	145	-	-	126	-	-	107	-	-	163	-	-
σ / kHz	4.3	-	-	4.3	-	-	2.9	-	-	4.7	-	-

Table S4. Comparison between experimentally determined and calculated rotational parameters of TFB-CH_4 and TFB -$(CH_4)_2$. *A*, *B*, and *C* are the rotational constants, μ_a, μ_b, and μ_c are the electric dipole-moment components. σ is the root-mean-square deviation of the fit, N is the number of rotational transitions included in the fit. The calculated spectroscopic constants of the minimum structures are optimized at the B3LYP-D3(BJ)/def2-TZVP level of theory.

	TFB-CH_4		Calc.	Diff. / %	TFB-$(CH_4)_2$			Calc.	Diff. / %
	A	E			AA	AE	EE		
A / MHz	1246.6338(11)	1245.07976(70)	1301	-4.2	1003.0910(18)	1002.37376(53)	1001.5310(10)	988	+1.5
B / MHz	1065.4930(18)	1059.95990(81)	1109	-4.2	789.4014(25)	788.84730(60)	788.29643(78)	836	-5.7
C / MHz	1003.3491(17)	1001.43451(82)	990	+1.2	713.2458(54)	710.12746(51)	707.09916(82)	750	-5.3
|Dipole comp.|	$\mu_b > \mu_c$, no μ_a		0.0/ 2.8/ 0.6	-	μ_c, no μ_a, no μ_b			0.0/ 0.0/ 2.8	-
N	19	44	-	-	13	37	23	-	-
σ / kHz	4.2	6.0	-	-	4.2	4.9	4.0	-	-

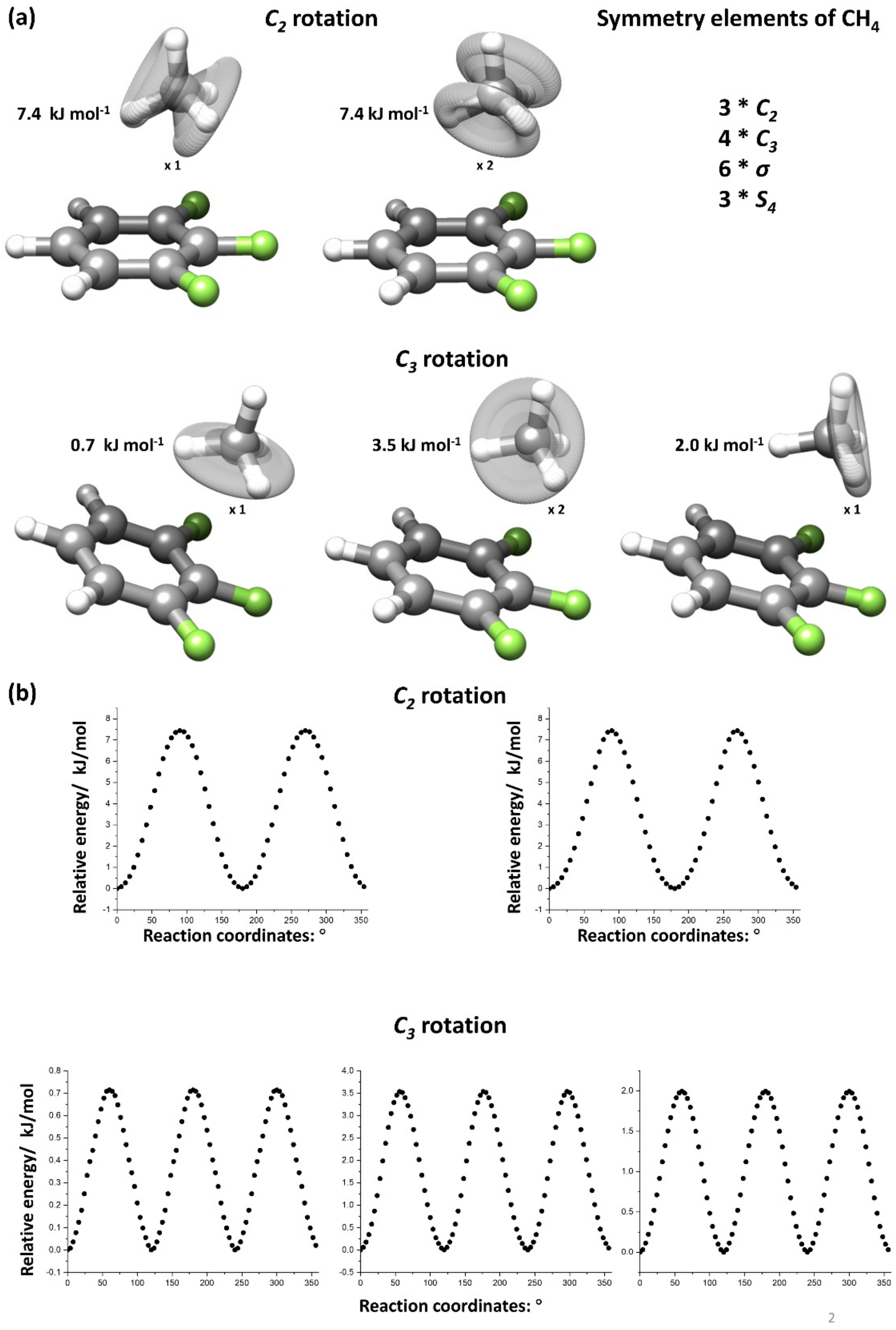

Figure S1. The possible internal rotation motions and energy surfaces of CH_4 on TFB. (a) Two internal rotation motions of CH_4 on TFB by the C_2 axes of CH_4, and three internal rotation motions of CH_4 on TFB by the C_3 axes of CH_4. (b) Potential energy scans of internal rotation motions of CH_4 on TFB at the B3LYP-D3(BJ)/def2-TZVP level of theory.

Table S5. The fitted rotational constants of TFB-CH_4 dimer ^{13}C isotopologues. The distortion constants are adopted from the parent TFB-CH_4 fitting.

	C1	C2	C3	C4	C7
A / MHz	1230.6624(10)	1238.3111(6)	1244.6810(6)	1243.4866(12)	1214.9080(9)
B / MHz	1059.9332(13)	1056.5445(6)	1056.5555(5)	1059.5821(21)	1039.0877(13)
C / MHz	992.1617(7)	994.1474(5)	998.6034(5)	1000.8213(9)	1000.5121(9)
N[b]	7	13	13	8	8
σ / kHz[b]	5.9	5.7	5.0	6.7	6.0

[a] The relative magnitude of the permanent dipole moment components, estimated from the experimental transition intensities.
[b] *N*: number of transitions in the fit, and *σ*: standard deviation in kHz.

Table S6. The Kraitchman coordinates of TFB-CH_4 dimer ^{13}C isotopologues with the sign adopted from the optimized minimum structure.

	C1	C2	C3	C4	C7
x / Å	0.0000(138)	1.21210(125)	1.19240(127)	0.0000(110)	0.0000(118)
y / Å	2.17667(70)	1.49578(101)	0.09992(1515)	-0.57092(269)	-0.56285(268)
z / Å	-0.18177(842)	-0.19114(794)	-0.35046(432)	-0.44287(347)	3.12992(49)

Table S7. The fitted rotational constants of TFB-CO_2 dimer ^{13}C isotopologues. The distortion constants are adopted from the parent TFB-CO_2 fitting.

	C1	C2	C3	C4	C5	C6	C7
A / MHz	1479.3608(350)	1472.7836(269)	1483.1940(181)	1487.3586(260)	1485.6008(257)	1487.5479(361)	1487.0366(179)
B / MHz	613.41323(79)	617.07865(59)	618.20326(38)	618.06868(54)	616.10878(59)	612.55513(71)	611.66961(35)
C / MHz	499.86585(75)	501.29820(56)	503.39585(34)	503.81431(48)	502.12577(49)	500.22548(73)	499.65065(34)
N[b]	12	15	14	12	12	14	14
σ / kHz[b]	7.7	6.8	4.4	5.8	4.9	8.1	4.0

[a] The relative magnitude of the permanent dipole moment components, estimated from the experimental transition intensities.
[b] *N*: number of transitions in the fit, and *σ*: standard deviation in kHz.

Table S8. The Kraitchman coordinates of TFB-CO_2 dimer ^{13}C with the sign adopted from the optimized minimum structure.

	C1	C2	C3	C4	C5	C6	C7
x / Å	2.51640(103)	1.29679(172)	0.22249(847)	0.44608(488)	1.72842(125)	2.74104(96)	-2.93833(64)
y / Å	-1.37972(190)	-1.91401(117)	-1.04117(181)	0.31544(690)	0.83206(261)	0.0000(423)	0.19810(952)
z / Å	0.49757(529)	0.05064(4473)	-0.39377(480)	-0.42808(509)	0.0000(445)	0.49591(534)	0.56745(332)

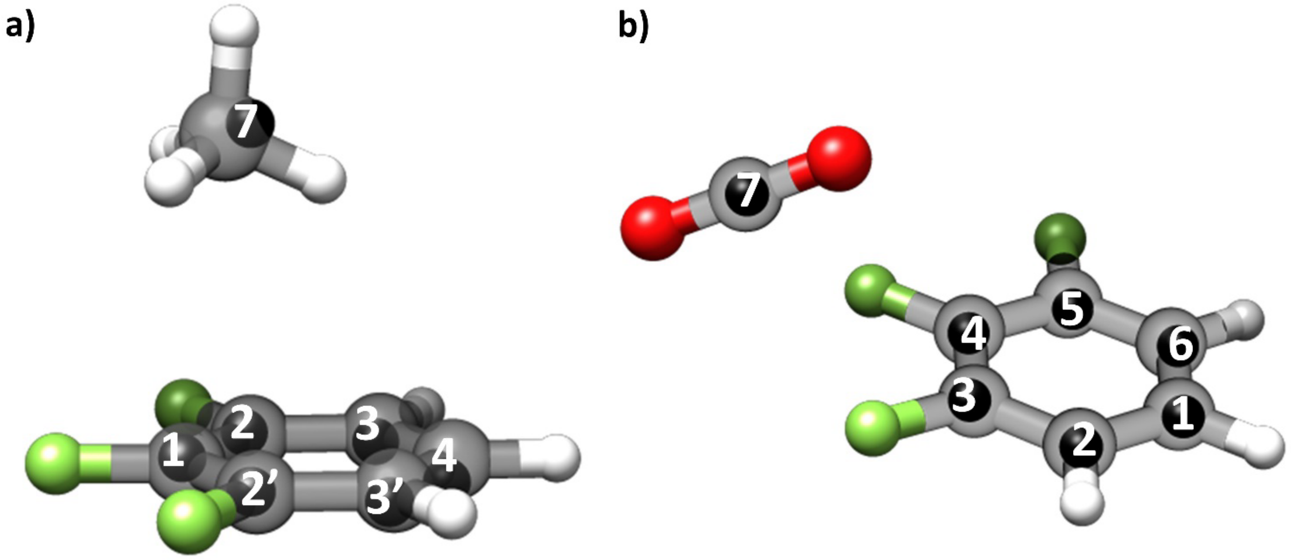

Figure S2. The Kraitchman structure and atom number label of TFB-CH_4 and TFB-CO_2 dimers.

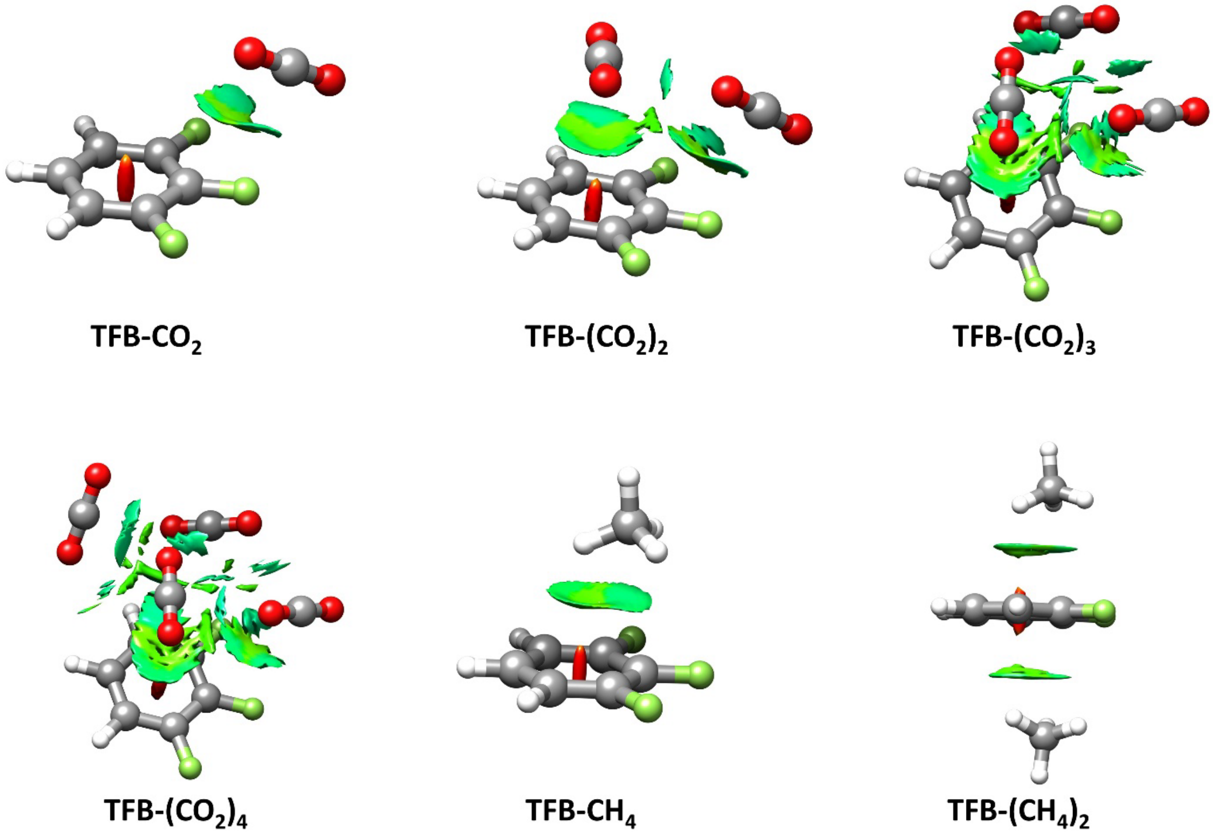

Figure S3. NCI plots of the observed TFB-$(CO_2)_n$ and TFB-$(CH_4)_m$ complexes.

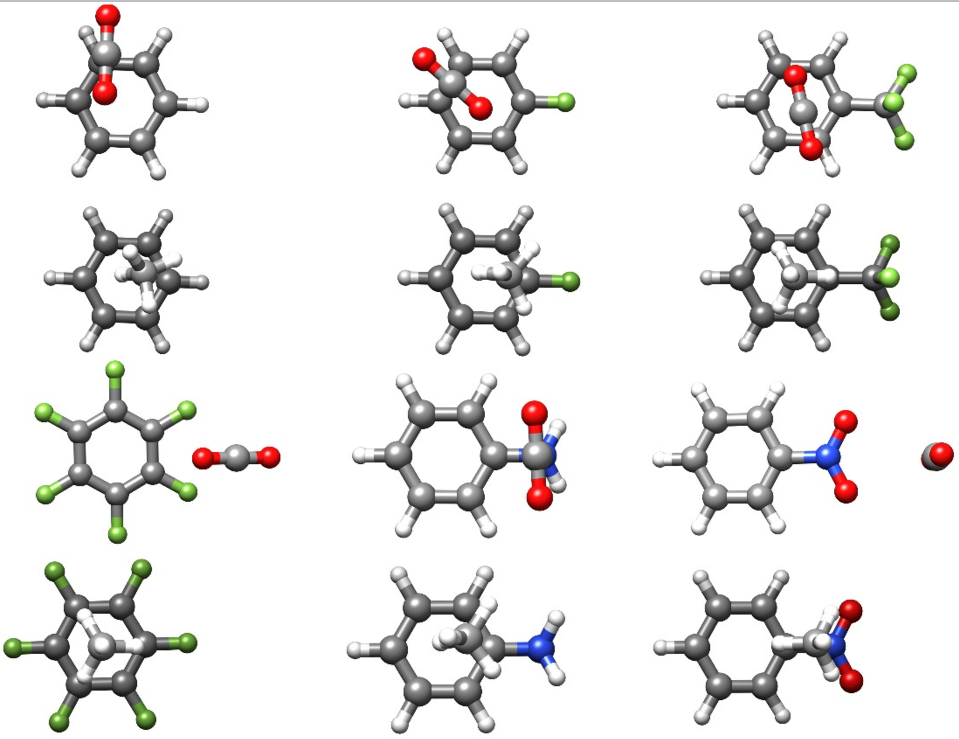

Figure S4. The optimized structures of representative aromatics-gas complexes, calculated at B3LYP-D3(BJ)/def2-TZVP level of theory.

Table S9 Binary binding energy decomposition of representative aromatics-gas complexes, calculated using SAPT2+3/aug-cc-pVDZ with δMP2 corrections in kJ/mol. The geometries correspond to the global minima of aromatics-gas binary complexes, each selected from their respective isomeric pools.

Binary complexes		$\Delta E_{Electrostatic}$		$\Delta E_{Induction}$		$\Delta E_{Dispersion}$		$\Delta E_{Exchange}$	ΔE_{total}	Ratio
benzene	CO_2	-9.3	34.1%	-1.9	7.1%	-16.0	58.7%	16.9	-10.3	1.8
	CH_4	-3.6	23.1%	-0.8	5.5%	-11.0	71.5%	9.8	-5.7	
1-fluorobenzene	CO_2	-9.0	33.3%	-1.8	6.7%	-16.2	59.9%	16.9	-10.1	1.7
	CH_4	-3.5	22.3%	-0.6	3.6%	-11.7	74.2%	9.8	-5.9	
TFB	CO_2	-8.2	37.6%	-1.1	5.1%	-12.5	57.3%	12.3	-9.5	1.4
	CH_4	-4.5	25.1%	-0.5	2.8%	-12.8	72.1%	10.9	-6.9	
hexafluorobenzene	CO_2	-7.2	35.2%	-1.0	5.1%	-12.1	59.6%	11.3	-9.0	1.3
	CH_4	-3.3	21.2%	-0.4	2.9%	-11.7	75.9%	8.7	-6.8	
trifluorotoluene	CO_2	-8.6	30.8%	-1.6	5.6%	-17.8	63.6%	16.5	-11.6	1.8
	CH_4	-3.5	21.1%	-0.5	3.0%	-12.5	75.8%	10.1	-6.4	
Aniline	CO_2	-19.0	48.8%	-2.3	6.0%	-17.6	45.2%	23.5	-15.4	2.2
	CH_4	-4.3	23.6%	-1.1	5.9%	-12.8	70.4%	11.3	-6.9	
nitrobenzene	CO_2	-16.1	53.2%	-1.6	5.3%	-12.6	41.5%	17.7	-12.6	1.6
	CH_4	-4.6	24.7%	-0.4	2.1%	-13.5	73.1%	10.7	-7.8	
